# Improved Productivity of Astaxanthin from Photosensitive *Haematococcus pluvialis* Using Phototaxis Technology

**DOI:** 10.3390/md20040220

**Published:** 2022-03-22

**Authors:** Kang Hyun Lee, Youngsang Chun, Ja Hyun Lee, Chulhwan Park, Hah Young Yoo, Ho Seok Kwak

**Affiliations:** 1 Department of Biotechnology, Sangmyung University, 20, Hongjimun-2Gil, Jongno-gu, Seoul 03016, Korea; oys7158@naver.com; 2 Department of Bio-Convergence Engineering, Dongyang Mirae University, 445-8, Gyeongin-ro, Guro-gu, Seoul 02841, Korea; youngsangchun@gmail.com; 3 Department of Convergence Bio-Chemical Engineering, Soonchunhyang University, 22, Soonchunhyang-ro, Asan-si 31538, Korea; jhlee84@sch.ac.kr; 4 Department of Chemical Engineering, Kwangwoon University, 20, Kwangwoon-ro, Nowon-gu, Seoul 01897, Korea

**Keywords:** *Haematococcus pluvialis*, astaxanthin, photosynthetic efficiency, microfluidics, photobioreactor

## Abstract

*Haematococcus pluvialis* is a microalgae actively studied for the production of natural astaxanthin, which is a powerful antioxidant for human application. However, it is economically disadvantageous for commercialization owing to the low productivity of astaxanthin. This study reports an effective screening strategy using the negative phototaxis of the *H. pluvialis* to attain the mutants having high astaxanthin production. A polydimethylsiloxane (PDMS)-based microfluidic device irradiated with a specific light was developed to efficiently figure out the phototactic response of *H. pluvialis*. The partial photosynthesis deficient (PP) mutant (negative control) showed a 0.78-fold decreased cellular response to blue light compared to the wild type, demonstrating the positive relationship between the photosynthetic efficiency and the phototaxis. Based on this relationship, the *Haematococcus* mutants showing photosensitivity to blue light were selected from the 10,000 random mutant libraries. The M1 strain attained from the phototaxis-based screening showed 1.17-fold improved growth rate and 1.26-fold increases in astaxanthin production (55.12 ± 4.12 mg g^−1^) in the 100 L photo-bioreactor compared to the wild type. This study provides an effective selection tool for industrial application of the *H. pluvialis* with improved astaxanthin productivity.

## 1. Introduction

The ketocarotenoid astaxanthin (3,3′-dihydroxy-β-carotene-4,4′-dione) straddles the cell membrane bilayer and acts as a super antioxidant that significantly reduces free radicals and oxidative stress [1,2,3]. Astaxanthin has been widely applied as a promising functional substance in the functional foods, pharmaceutical, cosmetics, and aquaculture industries due to its potential to prevent Alzheimer’s disease, atherosclerotic, and cardiovascular diseases [4,5,6,7]. The global astaxanthin market was estimated at USD 288.7 million in 2017 and forecasted to grow to USD 426.9 million in 2022 [8]. Astaxanthin can be produced synthetically or obtained from natural sources [9]. However, synthetic astaxanthin is not approved for human consumption directly by the Food and Drug Administration (FDA) as a food or dietary supplement due to several issues, such as food safety, potential toxicity in the final product, and sustainability [10]. Compared with synthetic astaxanthin, natural astaxanthin has various advantages such as high safety and antioxidant activity, environmental friendliness, sustainability, and renewability [11]. For these reasons, the demand for natural astaxanthin has increased.

Traditionally, natural astaxanthin has been produced from a variety of seafood, including shrimp, lobster, fish eggs, and microalgae [12,13,14]. Microalgae has advantages in astaxanthin production compared to other seafood in terms of productivity, production area, safety control, and stability [15]. Among the various microalgae, *Haematococcus pluvialis* is considered to be the most abundant source of natural astaxanthin (3–5% of dry biomass) [16]. In addition, natural astaxanthin obtained from *H. pluvialis* was confirmed as a food ingredient for humans by the European Commission (EC) and the European Food Safety Authority (EFSA) [17]. However, there are limitations to the utilization of natural astaxanthin on an industrial scale. Various studies have been actively conducted to improve astaxanthin productivity from *H. pluvialis* [16,18,19]. Astaxanthin productivity can be improved by maximizing the cell density in the green phase and by process optimization such as culture parameters and the design of photobioreactors in the red phase during *H. pluvialis* culture [16,18,19].

In order to improve astaxanthin productivity from *H. pluvialis*, various approaches have been used such as development of strains, optimization of culture conditions, protoplast fusion with other microalgae, and addition of nanoparticles [20,21,22,23]. Among these approaches, the development and selection of strains is the most important strategy in industrial production [24]. Random mutagenesis by ultraviolet (UV) irradiation is a common and cost-effective technique for strain development [25]. Various studies have been conducted to develop genetically modified mutants with high growth rate and astaxanthin productivity through random mutagenesis [26,27,28]. However, one of the disadvantages of random mutagenesis is that it is difficult to select strains with improved specific efficacy among numerous variants, and the selection process requires a lot of time and labor. Microfluidic-based technologies with specific light can rapidly screen strains at the single-cell level, taking into account the size, shape, and characteristics of microalgae [29]. In a previous study [30], strains with improved photosynthetic productivity were selected using a microfluidic device fabricated by applying the phototaxis technique.

**Figure 1:**
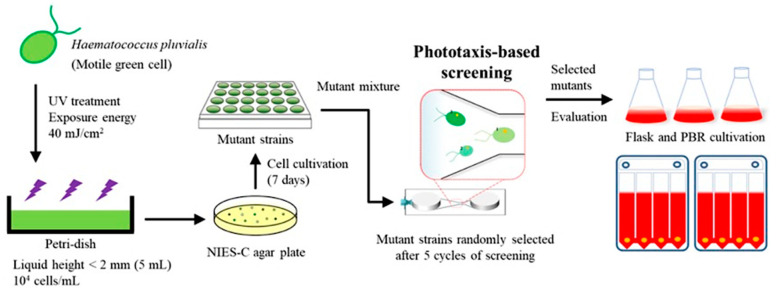
Schematic diagram of a phototaxis-based screening technique for the isolation of photosensitive strains using microfluidic devices. The screening was performed on a mixture of 10,000 mutants generated from the wild-type strain by UV random mutagenesis.

In this study, the *H. pluvialis* strain was developed with improved astaxanthin productivity by using the phototaxis technology and scaling it up using the photo-bioreactor for industrial application (Figure 1). At the micro scale, the phototactic response of *H. pluvialis* and the correlation between photosynthesis and phototaxis were investigated to select mutated *H. pluvialis* with high astaxanthin productivity. Mutants with high negative phototaxis selected by the microfluidic device were scaled up to a 100 L flat panel photo-bioreactor, and astaxanthin production was determined. This multi scale approach can be easily applied to various microalgae and provides an efficient selection method to develop the microalgal mutants with improved productivity of diverse metabolites such as the astaxanthin. Currently, most studies using the phototaxis properties of microalgae are related to photosynthetic efficiency. To the best of the authors’ knowledge, this is the first study utilizing phototaxis properties to screen *H. pluvialis* for improved astaxanthin productivity.

## 2. Results and Discussion

### 2.1. Investigation of Phototactic Properties of H. pluvialis

**Figure 2:**
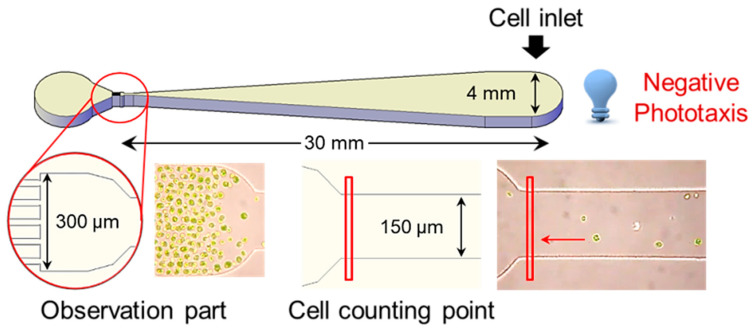
Microfluidic device design for detection of phototaxis of the *H. pluvialis*. Cells grown exponentially were loaded into an inlet chamber in the microfluidic device and exposed to blue LED (70 μmol photons m^−2^ s^−1^). The phototactic movements of cells were monitored under an inverted microscope and analyzed using video analyzing software.

Phototaxis is a reaction in which the direction of movement changes depending on the intensity of light [31]. Positive phototaxis indicates the characteristic of cells moving toward the light source at low-light intensity, whereas negative phototaxis indicates that the cell moves away from the light source at high-light intensity [32]. It is reported that the control of negative phototaxis is easier and more consistent than the positive phototaxis of cells [33]. To investigate the optical properties of the *H. pluvialis*, the cells, which were photo-autotrophically pre-cultured for 10 days, were diluted to a concentration of 10,000 cells mL^−1^, and then 50 µL aliquot (about 3000 cells) of the dilute solution was put into the inlet of the microfluidic device (Figure 2). Then, various light emitting diode (LED) lights (dark, blue, green, red, and white) were irradiated to the inlet part of the microfluidic device for 30 min to observe the negative phototaxis of the cells.

**Figure 3:**
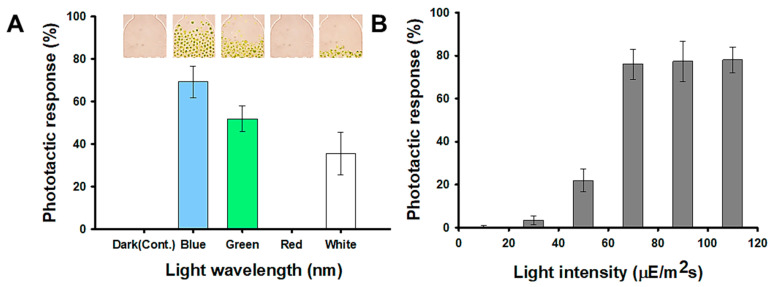
Light conditions for detecting phototaxis of *H. pluvialis*: (**A**) The phototactic responses of the wild type at five different light wavelength conditions; dark (control), blue (470 nm), green (540 nm), red (700 nm), white (all). Photo image showing the observation part of the microfluidic device after 30 min. (**B**) The phototactic responses of wild type at different light intensities; 10, 30, 50, 70, 90, 110 μmol photons m^−2^ s^−1^.

Figure 3A represents the negative phototaxis of the *H. pluvialis* to five different light wavelengths and *H. pluvialis* shows the most sensitive response to the blue light wavelength (470 nm). About 69.33% of the cells injected moved to the opposite chamber (observation part) for 30 min by the blue light. In the green light (540 nm), the phototactic response was reduced by 25% compared to the result in the blue light. In the red light (700 nm), there was no reaction to the light during 30 min as in the dark condition (control). These results show a trend similar to that of the model microalgae, *Chlamydomonas reinhardtii*, which has been reported to exhibit phototaxis by detecting the direction, intensity, and wavelength of light because it has an eyespot in the thylakoid membrane [30,34]. Tamaki et al. [35] reported that carotenoids, such as astaxanthin, fucoxanthin, and diadinoxanthin are major constituents of eyespot and are essential for the phototactic response. In addition, it has been reported that blue light significantly improves astaxanthin productivity of *H. pluvialis* [36]. Therefore, it can be inferred that *H. pluvialis*, which has sensitive phototaxis properties, contains a large amount of astaxanthin. Figure 3B is a result showing the light responses to different light intensities, and shows that the negative phototaxis of the *H. pluvialis* increased up to the 70 µmol photons m^−2^ s^−1^. However, there was little change at the intensity above 70 µmol photons m^−2^ s^−1^. Therefore, 70 µmol photons m^−2^ s^−1^ was set as the standard for measuring the phototactic behavior in this study.

### 2.2. Correlation between Photosynthesis and Phototaxis

**Figure 4:**
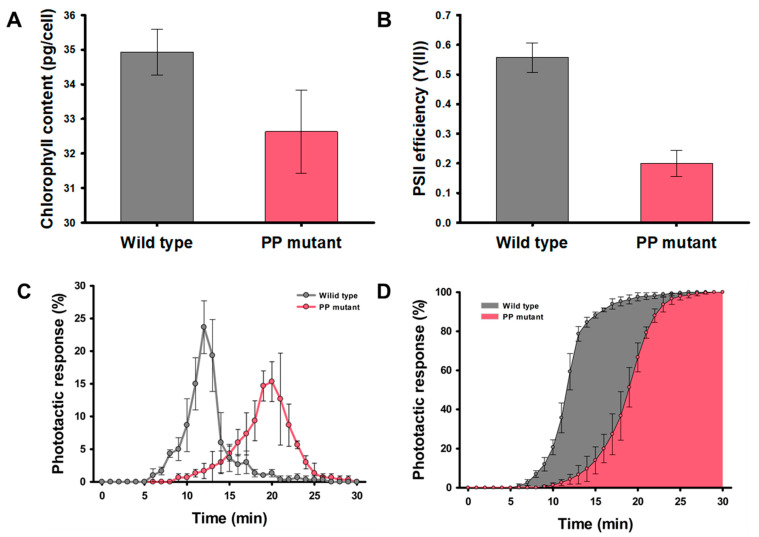
Optical characteristics of the wild type (grey) and PP mutant (red): (**A**) Chlorophyll content, and (**B**) Y(II) of the wild type and PP mutant. (**C**) The phototactic response, and (**D**) Cumulative histogram of phototactic response of the wild type and PP mutant for 30 min. The phototactic response was measured on 3000 cells per analysis. All data are the mean of three biological replicates.

To confirm the correlation between photosynthesis and phototaxis of the *H. pluvialis*, the partial photosynthesis deficient (PP) mutant was used as a negative control to compare with wild-type strain. The chlorophyll contents and the photosystem II (PSII) operating efficiency (Y(II)), indicating the photosynthetic efficiency of PP mutant, showed 6.7% and 64% less than that of wild type, respectively (Figure 4A,B). Hong et al. [26], reported that the total cell numbers of PP mutant in the green stage decreased by 32.2% compared to the wild-type strain because of partial photosynthesis deficient. These results are consistent with Wan et al. [37], who determined that *H. pluvialis* is suitable for mass culture on an industrial scale because it is easy to acquire large quantities of cells due to its high photosynthetic efficiency. Figure 4C,D represent the phototactic activities of the wild type and PP mutant to the blue light for 30 min. In the wild-type strain, 78.8% of the cells injected into the inlet of the microfluidic device reached the observation chamber 30 mm away from the inlet. The PP mutant showed 61.5% of the cells responded to the blue light, and it is 0.78-fold decrease compared to the wild-type strain. In addition, the cells that reached the opposite chamber took 12.4 min on average for the wild-type strain, but 19.1 min for the PP mutant. This means that the wild-type strain is highly reactivity to blue light, thereby increasing the cell migration rate. Figure 4D indicates the accumulative histogram of arriving cell numbers for 30 min. The average time required to reach 80% of the cells responding to the blue light was 13.3 min for the wild-type strain and more than 20 min for the PP mutant. These results indicate that the high Y(II) in the *H. pluvialis* is correlated with sensitive reactivity to the blue light. This positive correlation between phototaxis and photosynthetic efficiency was also proved in model microalgae, *C. reinhardtii* [30]. 

### 2.3. Strain Selection Using the Microfluidic Device and Flask Cultivation

The microfluidic device was fabricated using polydimethylsiloxane (PDMS) to isolate mutated *H. pluvialis* with high photosynthetic efficiency and astaxanthin productivity based on the relationship between phototaxis and the photosynthetic efficiency. PDMS is useful for cell analysis using a microfluidic system because it is possible to efficiently monitor the cell movements and change in cell physiology at the single cell level due to its transparent property [38,39]. Ten thousand mutant libraries of the *H. pluvialis* were constructed by random mutagenesis using ultraviolet rays (Figure 1). After collecting cultures of all mutants, the screening process was repeated up to five times to increase the strains exhibiting a rapid phototactic response from a mixture of 10,000 mutants. After five screening cycles, a mixture of mutants showing a rapid reactivity to the blue light was obtained. The mixture was plated on an agar plate for isolating each strain as separated colonies. Next, 10 strains were selected from agar plates and cultured, and photoautotrophic growth was analyzed. The M1 showing the highest growth and astaxanthin production among 10 mutant strains were selected for further evaluation of their performances. 

**Figure 5:**
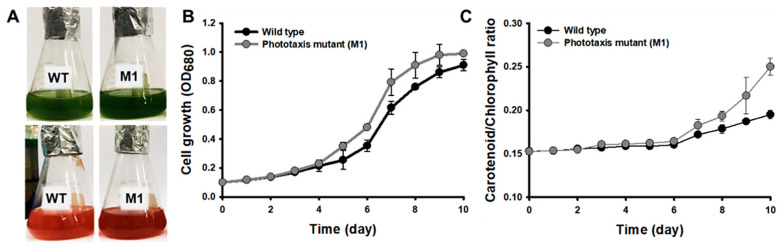
Flask cultivation of the wild type and M1 mutant screened by phototaxis: (**A**) Photo image of the wild type and M1 mutant under growth (up) and induction stage (down). (**B**) Absorbance in growth stage, and (**C**) Carotenoid/Chlorophyll ratio of in induction stage. The cells were grown in NIES-C medium and CO_2_ concentration and light intensity were fixed at 5% and 40 µmol photons m^−2^ s^−1^, respectively. Error bars indicate the standard deviations of the means for three experiments.

The wild-type strain and M1 mutant were inoculated into the flask for two stage cultivation (growth stage and induction stage) for 20 days (Figure 5A). In the growth stage, the M1 mutant showed faster and higher growth compared to the wild-type strain (Figure 5B). The maximum specific growth rate (µ_max_) was calculated from the growth curve. The µ_max_ of M1 mutant was 0.702 h^−1^, which was 1.17-fold higher than µ_max_ of the wild-type strain (0.598 h^−1^). The Y(II) of the M1 mutant during the growth stage showed 0.63 ± 0.012, which was 13% higher than that of the wild type, suggesting that rapid phototactic response to the blue light can be helpful to improve Y(II) [30]. After 10 days of growth, the NIES-C medium was replaced with the nitrogen-deficient NIES-N medium because nitrogen deficiency enhances astaxanthin productivity of *H. pluvialis* [40]. Additionally, light intensity increased up to 200 µmol photon m^−2^ s^−1^ to induce the astaxanthin biosynthesis by oxidative stresses [41]. Under high light intensity, reactive oxygen species (ROS) are accumulated in microalgae by the over-reduction of the photosynthetic electron transport chain [42]. Microalgae produce carotenoids including astaxanthin as a defense mechanism against oxidative stress such as ROS [43]. Figure 5C shows the change in the ratio of carotenoid and chlorophyll according to the synthesis of the astaxanthin in the induction stage. It shows that the conversion to carotenoid occurs more efficiently in the M1 mutant than in the wild-type strain according to the environmental change. This is because the astaxanthin synthesis was rapidly conducted as a defense mechanism against oxidative stress caused by high light intensity. The productivity of astaxanthin of the M1 mutant showed 171.6 ± 4.16 mg L^−1^, which was 1.26-fold higher than that of the wild-type strain.

### 2.4. Validation of the M1 Mutant Performance in Large Scale Cultivation

**Figure 6:**
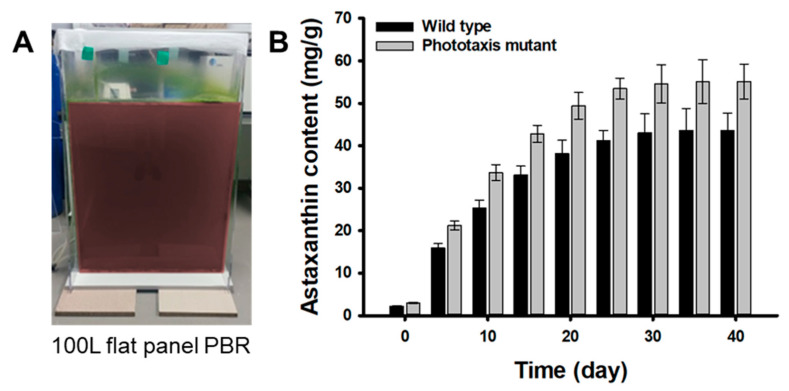
Large scale cultivation of the wild type and the M1 mutant: (**A**) Photo image of the 100 L flat panel photo-bioreactor. (**B**) Astaxanthin content of the wild type and M1 mutant. Cells suspensions were aerated at a flow rate of 0.1 vvm with 5% CO_2_-enriched air. Error bars indicate the standard deviations of the means for three experiments.

In order to confirm the improved astaxanthin productivity of the M1 mutant, the flat panel photo-bioreactor of 100 L scale was manufactured using acrylic. The M1 mutant and wild-type strain pre-cultured in a 1 L flask for 10 days were inoculated and grown for 30 days (Figure 6A). In the growth phase, the M1 mutant showed a faster growth rate and 1.1-fold higher biomass production than the wild-type strain, which was similar to the results of the flask experiment (data not shown). This is because the M1 mutant has a higher photosynthetic efficiency than the wild type for the same light intensity condition (40 µmol photons m^−2^ s^−1^). After the vegetative growth phase, cells were exposed to a continuous strong light at 200 μmol photons m^−2^ s^−1^ along with N-deprivation for the next 40 days in the red stage. The astaxanthin contents formed in the M1 mutant cells through an induction period of 40 days was 55.12 ± 4.12 mg g^−1^, which was 1.26-fold higher than that of the wild-type strain (43.62 ± 3.98 mg g^−1^) (Figure 6B). These results show that the M1 mutant has a 32% higher light reactivity and 18.5% faster light response rate than the wild-type strain, so it could rapidly sense the light faster than the wild-type strain against strong light irradiation during the induction period. Therefore, it is inferred that the M1 mutant promoted the synthesis of the astaxanthin to prevent oxidation of cells faster than the wild-type strain.

Various mutagenesis and isolation strategies to enhance astaxanthin productivity from *H. pluvialis* are summarized in Table 1. 

As a mutagenesis strategy, mainly UV irradiation and ethyl methane sulfonate (EMS) treatment were performed, and approaches such as γ–ray irradiation and dielectric barrier discharge (DBD) plasma irradiation were also carried out. As a strategy to isolate mutant strains, most studies have screened resistant strains using chemicals such as diphenylamine (DPA), nicotine, and azide as inhibitors. Wang et al. [20] developed strain *H. pluvialis* DPA12–1 with an astaxanthin content of 47.2 mg g^−1^, 1.7-fold higher than that of the wild type, through UV irradiation and ethyl methane sulfonate (EMS) treatment. Chen et al. [44] reported that the astaxanthin content of *H. pluvialis* EU3, mutated by UV irradiation and EMS treatment and isolated by nicotine, improved to 25.0 mg g^−1^, 1.3-fold higher than that of wild type. *H. pluvialis* H2–419–4 [47] mutated by UV irradiation and EMS treatment showed that astaxanthin production and growth rate were improved by 1.3-fold and 1.7-fold, respectively, compared to the wild type. Hong et al. [46] developed *H. pluvialis* M13, which showed 1.6-fold higher astaxanthin production than wild type by isolation using azide after UV irradiation. Cheng et al. [47] induced CO_2_ stress in *H. pluvialis* mutated by γ–ray irradiation and astaxanthin production reached 70.8 mg g^−1^, a 2.4-fold improvement compared to wild type. *H. pluvialis* M3 [48] was mutated by DBD plasma irradiation and isolated using DPA. Astaxanthin production of M3 mutant was 33.5 mg g^−1^, which was 1.5-fold higher than that of wild type. Astaxanthin production of *H. pluvialis* B24 [49] isolated using DPA after mutation by EMS was found to be 26.4 mg g^−1^. The growth rate of the mutant was slightly higher or comparable to that of the wild type. *H. pluvialis* M1 developed in this study showed that astaxanthin production (55.12 ± 4.12 mg g^−1^) and growth rate were improved about 1.3-fold and 1.2-fold, respectively, compared to wild type. These results prove that the M1 mutant, selected using the microfluidic device, has similar or higher astaxanthin content than other mutants by UV irradiation. Although many studies have successfully developed H with improved astaxanthin productivity, chemicals that can cause environmental pollution, such as EMS, DPA, and nicotine, were used in the isolation process. Our strain selection strategy has the advantage of being able to efficiently screen strains with improved astaxanthin productivity in an eco-friendly approach.

## 3. Materials and Methods

### 3.1. Algal Strains and Culture Conditions

*Haematococcus pluvialis* NIES-144 (wild type) was obtained from the National Institute for Environmental Studies (Tsikuba, Japan). The PP mutant strain developed by chemical mutagenesis was used for determining whether differences in the photosynthetic efficiency affect the phototaxis [26]. The *H. pluvialis* was cultured by two-stage culture strategy; the ‘green’ stage for vegetative growth and the ‘red’ stage for the astaxanthin production. The cells were photo-autotrophically cultured in NIES-C medium (pH 7.5) with the vegetative growth at the low light intensity (40 µmol photons m^−2^ s^−1^ with a continuous light) for 10 days. After that, the astaxanthin accumulation was induced by the nutrient starvation with NIES-N medium (nitrogen deficient NIES-C medium, pH 7.5) at the strong light intensity (200 μmol photons m^−2^ s^−1^ with a continuous light) for 20 days in the red stage [50]. The light was provided by cool white LED lamps and the light intensities were measured by a LI-250 quantum photometer (Lambda Instrument, Blacksburg, VA, USA). The vegetative cells, with an initial cell density of 0.1 (OD_680_), were inoculated into 100 mL Erlenmeyer flasks for the lab scale cultivation and 100 L flat panel photo-bioreactor, which was made of acrylic, for the large scale cultivation, respectively. All cell suspensions were aerated at a flow rate of 0.1 vvm with 5% CO_2_-enriched air at 23 °C.

### 3.2. UV Irradiation Procedure for Mutagenesis

The wild-type cells (NIES-144) in the exponential phase experiencing the dark period were used for UV mutagenesis to improve transformation efficiency as described previously [51]. The cells diluted to 10,000 cells mL^−1^ were spread on an NIES-C agar plate, and were irradiated using 254 nm UVLS-225D Mineralight UV Display lamp (UVP, Upland, CA, USA), with the intensity of 0.02 mW cm^−2^. UV intensities were measured by GT-510 UV meter (Giltron, Norwood, MA, USA). The survival rates of UV-treated cells depending on the UV energy were calculated by counting the colonies on solid agar plates under the different exposure times: 0 min (0 mJ cm^−2^; control), 16 min (20 mJ cm^−2^), 32 min (40 mJ cm^−2^), 48 min (60 mJ cm^−2^), and 60 min (80 mJ cm^−2^). The mutant libraries were made under optimal conditions of 40 mJ cm^−2^ of UV energy (32 min of UV exposure time), representing about 2.6% of survival rates, and were kept for the regeneration during 24 h in the dark condition.

### 3.3. Fabrication of Microfluidic Device

A PDMS-based microfluidic device was fabricated using standard soft photolithography [52]. The design of the microstructure was generated using AutoCAD software and printed on transparent photomask film. A silicon mold master was fabricated by using SU-8 negative photoresist (SU-8 50, Microchem, Newton, MA, USA) on silicon wafers. The PDMS pre-polymer (10:1 mixture of 184 Sylgard base and curing agent, Dow Corning) was poured on the SU-8 mold and cured thermally at 80 °C. The PDMS layer containing the microchannel was bonded to a glass slide using oxygen plasma. The microfluidic device was composed of an inlet chamber (4 mm in diameter), outlet chamber (4 mm in diameter), and microchannel (30 mm in length, 50 μm in height) including observation zone (channel length 300 μm, width 100 μm, height 50 μm) near the outlet chamber. The width of the microchannel gradually decreased from 4 mm (at the inlet) to 150 μm (at the observation zone) to reduce hindrance of the phototactic movement by microchannel and facilitate the monitoring of the phototactic response at the single-cell resolution level (Figure 2).

### 3.4. Analysis of Phototactic Response in Microfluidic Device

Cells were grown for 10 days photo-autotrophically under continuous light at a light intensity of 40 μmol photons m^−^^2^ s^−^^1^. The culture was then diluted to a density of 40,000 cells mL^−^^1^ and dark-adapted for 30 min to make cells sensitive to light stimulus. A 50 μL aliquot of the diluted culture was loaded into the inlet chamber of the microfluidic device, and an equal volume of NIES-C medium was loaded into the outlet chamber. Cells were collected into the inlet chamber via exposure to blue LED light at the end of the outlet chamber. After hydrostatic balance was established in the microchannel, a blue LED (70 μmol photons m^−^^2^ s^−^^1^) was illuminated at the end of the inlet chamber to evoke negative phototaxis. The phototactic movements of cells were monitored and recorded under an inverted microscope (Olympus CKX41, Tokyo, Japan) equipped with a digital video camera (Canon EOS 700D, Tokyo, Japan). Cells arriving at the observation zone near the outlet chamber were counted, and their arrival times were automatically recorded for 30 min using custom software.

### 3.5. Phototaxis-Based Screening

Each colony on agar plates was transferred into 1 mL of NIES-C medium in each well of a 24-well microplate and cultured under low light condition (40 μmol photons m^−^^2^ s^−^^1^) at 23 °C. All cultures of 10,000 transformants were then mixed, centrifuged, and resuspended in 6 mL of NIES-C medium to a cell density of 6.0 × 10^8^ cells mL^−^^1^. For screening of the mixture of 10,000 transformants, a microfluidic device with enlarged chambers (8 mm in diameter) and a microchannel (width from 8 mm to 0.4 mm, height 100 μm) was used. A 2.75 mL aliquot of dark-adapted cell mixture was loaded into the inlet chamber and exposed to blue LED light (70 μmol photons m^−^^2^ s^−^^1^) for 10 min to isolate cells showing fast phototaxis. The cells arrived at the outlet chamber within 10 min and were collected by pipetting and recovered in NIES-C medium for 2 h. The cells were then used for the next cycle of phototaxis-assisted screening. After five cycles of screening, we obtained the cell mixture, and a 100 μL aliquot of the culture was plated onto NIES-C agar for the isolation of mutants as separated colonies. To compare the growth of mutant mixtures between each cycle of phototaxis-based screening, cells were inoculated to a density of 10,000 cells mL^−^^1^ and grown photo-autotrophically.

### 3.6. Analytical Methods

Chlorophyll fluorescence was measured with an FMS2 fluorometer (Hansatech, Norfolk, UK). Cells grown to an exponential phase (30 μg of chlorophyll a) were loaded onto a glass-fiber filter, and the filter was placed on the leaf clip. For determination of the PSII operating efficiency (Y(II)), cells (without dark-adaptation) were exposed to stepwise-increasing actinic light (from 1 to 900 μmol photons m^−^^2^ s^−^^1^) for 20 s at each light intensity, and a saturating flash (3000 μmol photons m^−^^2^ s^−^^1^, 0.7 s duration) was applied to measure Fm’. Y(II) was calculated as (Fm’–Fs)/Fm’. The cellular chlorophyll and carotenoid contents were determined spectrophotometrically as described [53,54]. 

The cell density was determined by measuring OD_680_ using UV-spectrophotometer (Shimadzu, Kyoto, Japan) or dry cell weight (DCW) every 24 h. DCW was only determined for large-scale cultivation by filtering aliquots of samples using pre-weighed filter paper. Then, the cell suspensions (10 mL) were filtered with GF/F glass microfiber filters (Whatman, Cambridge, UK) and dried at 105 °C overnight. DCW was determined by the difference between the mass of the biomass-containing filter paper and that of pre-weighed filter paper.

In order to identify the accurate amount of intracellular astaxanthin using a high performance liquid chromatography (HPLC) system, algal cells were collected by centrifuging the culture fluid at 3000 rpm for 10 min at 4 °C, and the astaxanthin in cell pellet was extracted using a homogenizer (TissueLyser II, Qiagen, Hilden, Germany) in the presence of methanol and glass beads [55]. Then, the homogenized lysates were saponified with 0.01 M KOH to convert the esterified astaxanthin into free forms.

After saponification, the astaxanthin concentration of each sample was determined by HPLC system equipped with two LC-10AD pumps and SPD-10 UV-Vis detector (Shimadzu, Kyoto, Japan). The extracts were separated using a 250 × 4.6 mm HS-303 hydrosphere C_18_ column (YMC, Kyoto, Japan). The mobile phase consisted of solvents A (dichloromethane: methanol: acetonitrile: water, 5.0: 85.0: 5.5: 4.5, *v/v*) and solvent B (dichloromethane: methanol: acetonitrile: water, 22.0: 28.0: 45.5: 4.5, *v/v*). For the effective separation of astaxanthin, a linear gradient system was used: 0% B for 8 min, a linear gradient from 0 to 100% B for 12 min, and 100% B for 50 min. The flow rate was set as 1.0 mL min^−1^ and the peaks were measured at 480 nm [50].

## 4. Conclusions

In this study, we screened mutant strains with photosensitivity using the negative phototaxis of the *H. pluvialis* in microfluidic device. It was confirmed that the more sensitive the mutant, the higher the astaxanthin productivity. In addition, large-scale cultivation in 100 L photo-bioreactor shows that the strains selected through phototaxis technology can be used in a more scaled-up culture for commercialization. We are planning follow-up studies to screen more cells faster and more efficiently. This screening strategy using the phototaxis of microalgae is advantageous in securement of *H. pluvialis* with improved growth rate and astaxanthin productivity.

## Figures and Tables

**Table 1:** Summary of strain development strategies for improved astaxanthin productivity from *H. pluvialis*.

Strain	Mutagenesis Strategy	Isolation Strategy	Astaxanthin Production		Growth Rate Improvement	Ref
			Unit	Improvement		
DPA12–1	UV, EMS ^1^	DPA ^2^	47.2 mg g^−1^	1.7-fold	1.4-fold	[20]
EU3	UV, EMS	Nicotine	25.0 mg g^−1^	1.3-fold	Similar	[44]
H_2_–419–4	UV, EMS		37 pg cell^−1^	1.3-fold	1.7-fold	[45]
M13	UV	Azide	174.7 mg L^−1^	1.6-fold	Similar	[46]
Not specified	γ–ray		70.8 mg g^−1^	2.4-fold	1.2-fold	[47]
M3	DBD ^3^ plasma	DPA	33.5 mg g^−1^	1.5-fold	1.6-fold	[48]
B24	EMS	DPA	26.4 mg g^−1^	1.3-fold	1.6-fold	[49]
M1	UV	Phototaxis	55.1 mg g^−1^	1.3-fold	1.2-fold	This study

^1^ EMS: ethyl methane sulfonate; ^2^ DPA: diphenylamine; ^3^ DBD: dielectric barrier discharge.

## Data Availability

The data presented in this study are available on request from the corresponding author.
